# Association of long-term treatment outcomes with changes in PET/MRI characteristics and the type of early treatment response during concurrent radiochemotherapy in patients with locally advanced cervical cancer

**DOI:** 10.1007/s00066-025-02389-w

**Published:** 2025-03-31

**Authors:** Radovan Vojtíšek, Jan Baxa, Petr Hošek, Petra Kovářová, Martin Vítovec, Emília Sukovská, Jan Kosťun, Pavel Vlasák, Jiří Presl, Jiří Ferda, Jindřich Fínek

**Affiliations:** 1 Faculty of Medicine, Charles University, Pilsen, Czech Republic; 2 Department of Oncology and Radiotherapy, University Hospital in Pilsen, alej Svobody 80, 304 60 Pilsen, Czech Republic; 3 Department of Imaging Methods, University Hospital in Pilsen, alej Svobody 80, 304 60 Pilsen, Czech Republic; 4 Biomedical Center, Faculty of Medicine in Pilsen, Charles University, alej Svobody 76, 323 00 Pilsen, Czech Republic; 5 Department of Gynecology and Obstetrics, University Hospital in Pilsen, alej Svobody 80, 304 60 Pilsen, Czech Republic

**Keywords:** Uterine cervical neoplasms, Brachytherapy, Survival, Positron-emission tomography, Magnetic resonance imaging

## Abstract

**Purpose:**

We aimed to find predictive tumour characteristics as detected by interim positron-emission tomography/magnetic resonance imaging (PET/MRI) in cervical cancer patients. We also investigated the type of interim response. Furthermore, we compared the investigated parameters with disease-free (DFS) and overall survival (OS) outcomes.

**Methods:**

We evaluated 108 patients treated between August 2015 and January 2023 with external-beam radiotherapy (EBRT) and image-guided adaptive brachytherapy (IGABT) who had undergone pretreatment staging, subsequent mid-treatment evaluation after completed EBRT and definitive restaging 3 months after completing the whole treatment using PET/MRI. Patients were then divided into two groups based on the RECIST and PERCIST criteria: responders (achieving complete metabolic response, CMR) and non-responders (non-CMR). These two groups were compared using selected parameters obtained at pre-PET/MRI and mid-PET/MRI. The early response to treatment as evaluated by mid-PET/MRI was categorized into three types: interim complete metabolic response, interim nodal response and interim nodal persistence.

**Results:**

Mid-TLG‑S (the sum of total lesion glycolysis for the primary tumour plus pelvic and para-aortic lymph nodes) parameter showed the best discriminatory ability for predicting non-CMR. The second factor with significant discriminatory ability was mid-MTV‑S (the sum of the metabolic tumour volume of the primary tumour plus pelvic and para-aortic lymph nodes). The strongest factor, mid-TLG‑S, showed a sensitivity of 40% and a specificity of 90% at a threshold value of 70. We found a statistically significant association of DFS and OS with the following parameters: number of chemotherapy cycles, early response type and CMR vs. non-CMR.

**Conclusion:**

We were able to identify thresholds for selected parameters that can be used to identify patients who are more likely to have worse DFS and OS. The type of early response during concurrent chemoradiotherapy (CCRT) was also significantly associated with DFS and OS. These aspects represent an important contribution to the possible stratification of patients for subsequent individualised adjuvant treatment.

## Purpose

Cervical cancer is the fourth most common cancer in women and the eighth most common cancer overall, with an estimated 662,000 new cases worldwide in 2022 [1]. Concurrent chemoradiotherapy (CCRT) is the standard treatment approach in patients with locally advanced cervical cancer (LACC) [1].

Currently, the best way to determine the exact extent of disease is to use integrated positron-emission tomography/magnetic resonance imaging (PET/MRI) [3]. With this examination, metabolic, functional and morphological parameters can be determined in a single examination, thus reflecting different aspects of tumour biology. This combination of detected parameters can be very valuable for predicting the prognosis of patients [4]. Quantitative multiparametric PET/MRI is also used to monitor the effect of initial treatment in patients with LACC, allowing for more advanced assessment of the treatment response [4].

Despite the large number of long-term complete remissions after CCRT combining external-beam radiotherapy (EBRT) and image-guided adaptive brachytherapy (IGABT) in patients with LACC, there is still a nonnegligible incidence of local and distant relapses, the further therapeutic management of which is very challenging and limited. Therefore, it is extremely important to identify early those patients who are more likely to relapse and who could benefit from treatment intensification, most likely in the form of adjuvant chemotherapy (ChT).

In 2021, we published a paper showing that PET/MRI performed during CCRT is capable of providing metabolic and anatomical parameters that have discriminatory power for predicting failure to achieve a complete metabolic response (CMR) [6, 7].

The aim of the current study was to follow up our previous study and to evaluate the predictive value of metabolic (18F-fluorodeoxyglucose [FDG] uptake parameters), molecular (diffusion-weighted imaging parameters) and anatomical tumour characteristics detected by interim PET/MRI at the end of EBRT/CCRT in a larger cohort of patients with longer follow-up. In addition, we investigated a new important predictive parameter: the type of interim response. We also compared all the investigated parameters with survival outcomes.

## Materials and methods

### Patients

After we had started to use IGABT, between May 2012 and April 2024, 183 patients suffering from histologically proven cervical cancer and not suitable for surgery underwent radical radiotherapy (RT) or CCRT at the Department of Oncology and Radiotherapy, University Hospital Pilsen, Czech Republic. Of this cohort, we analysed only patients who underwent pretreatment staging, subsequent mid-treatment evaluation after completed EBRT and definitive restaging 3 months after completing the whole treatment, in each case using PET/MRI. A total of 108 patients met these criteria, all of whom were treated at our department between August 2015 and January 2023. In the period preceding August 2015, we had used only MRI or PET/CT, as PET/MRI was not yet available in our hospital.

The median age of the analysed patients was 57 years (range 32–83 years). Almost half of the patients (48; 44.5%) had been diagnosed at stage IIIC1 according to the 2018 International Federation of Gynecology and Obstetrics (FIGO) classification [8]. Thirty patients (27.8%) were diagnosed at stage IIB, 22 patients (20.4%) at stage IIIC2 and 5 patients (4.6%) at stage IVB. One patient (0.9%) each had stage IB1, IIIB and IVA (Table 1). All patients signed an informed consent form prior to any treatment modality and any imaging procedure.

**Table 1:** Patient, tumour and treatment characteristics. The medium age of the patients was 57 (range 32–83)

Histology (no. patients; %)	Squamous cell carcinoma	103	95.4%
	Adenocarcinoma	5	4.6%
Grade (no. patients; %)	X	12	11.1%
	1	16	14.8%
	2	56	51.9%
	3	24	22.2%
FIGO stage (no. patients; %)	IB1	1	0.9%
	IIB	30	27.8%
	IIIB	1	0.9%
	IIIC1	48	44.5%
	IIIC2	22	20.4%
	IVA	1	0.9%
	IVB	5	4.6%
Chemotherapy (no. cycles; cisplatin 40 mg/m^2^ weekly)	No ChT	15	13.9%
	1	1	0.9%
	2	11	10.2%
	3	20	18.5%
	4	22	20.4%
	5	34	31.5%
	6	4	3.7%
	7	1	0.9%
**–**	< 5	69	63.9%
	≥ 5	39	36.1%
Nodal involvement (no. patients; %)	N0	32	29.6%
	N1	50	46.3%
	N2	26	24.1%

*FIGO* International Federation of Gynecology and Obstetrics, *ChT* chemotherapy

### External-beam radiotherapy and brachytherapy planning, chemotherapy

In all 108 patients (100%), EBRT was combined with brachytherapy (BT). All patients underwent pretreatment staging with PET/MRI before RT or CCRT was initiated. EBRT planning was based on a contrast-enhanced CT scan of the abdomen and pelvis of patients in a supine position. Treatment planning as well as the treatment itself were performed with full bladder and empty rectum. We used either intensity-modulated radiotherapy/volumetric modulated arc therapy (IMRT/VMAT; 33 patients, 30.5%) or IMRT/VMAT with a simultaneous integrated boost (IMRT/VMAT-SIB; 75 patients, 69.4%). All patients were treated with pelvic EBRT. Patients received extended-field RT that included the paraaortic lymph node regions if lymph node involvement in either the paraaortic or pelvic nodal chains had been confirmed. The prescribed dose of EBRT was 45 Gy. In the presence of lymphadenopathy, the IMRT/VMAT-SIB technique was used with two dose levels of 45 Gy or 55 Gy in 25 fractions. Lymphadenopathy was not confirmed by biopsy in any patient. Two patients had small nodal involvement in the lower mediastinum (and this area was part of the target volume), and one patient each had metastases in the left supraclavicular area (which regressed after ChT), right ovary (part of the target volume) and rectovaginal septum (also part of the target volume). The IMRT and the VMAT plans were normalized to cover ≥ 99% of the planning target volume (PTV) with 90% of the prescription dose and ≥ 95% of the PTV with 100% of the prescription dose. Plans were optimized in the Monaco® treatment planning system (Elekta, Stockholm, Sweden). All sets of plans were created using the same 10-MV photon beams from an Elekta Synergy linear accelerator equipped with an 80-leaf multileaf collimator. Patients were treated using either IMRT plans with nine coplanar fields using equally spaced gantry angles or with VMAT plans, which were performed using two coplanar arcs.

In 93 cases (86.1%), EBRT was combined with concomitantly administered chemotherapy (ChT) with cisplatin 40 mg/m^2^ weekly (Table 1). However, most patients did not receive all possible cycles of chemotherapy, mainly due to haematological toxicity and incipient renal insufficiency.

Immediately after the end of EBRT, BT was applied with a total of four planned applications. In all patients, we applied 3 T-MRI-based image-guided adaptive BT (IGABT) with the uterovaginal applicators Vienna Ring CT/MR, Interstitial Ring CT/MR or Venezia applicator (Elekta); the treatment planning systems Oncentra Masterplan® and Oncentra Brachy® (Elekta); and HDR (high-dose-rate) after-loading machines MicroSelectron® and Flexitron® (Elekta) with an iridium (^192^Ir) source. The whole procedure and treatment planning were performed according to the recommendations issued by the Gynaecological Working Group of the Groupe Européen de Curiethérapie (GEC) and the European Society for Radiotherapy and Oncology (GEC-ESTRO) published in 2005 and 2006 [9, 10] and also adopted by the American Brachytherapy Society (ABS) [11, 12]. The prescribed dose was 7 Gy to the 100% isodose. The coverage goal was quantitatively evaluated by the high-risk clinical target volume (HR-CTV) D_90_ (the dose covering 90% of the HR-CTV), which should ideally exceed 100% of the prescription dose. All doses were reported as the equivalent dose corresponding to the conventional fractionation using 2 Gy per fraction (EQD2). The linear quadratic model with values of α/β = 10 for the tumour and α/β = 3 for organs at risk was used for the dose recalculations, as described in detail in our previous publication regarding clinical outcomes and late side effects of 3D BT in cervical cancer in our institution [13, 14].

### PET/MRI protocol

The PET/MRI scans were obtained using an integrated PET/MRI scanner (Biograph mMR, Siemens Healthineers, Erlangen, Germany). Patients were advised to abstain from food for a duration of 6 h, and their blood glucose levels were measured to verify values below 150 mg/dL. Eligible patients received the radiopharmaceutical 18F-FDG with an activity of 2.5 mBq/kg. Following a 60-minute accumulation interval, PET images and MRI data were simultaneously acquired.

In the initial phase, a comprehensive diagnostic MRI examination of the pelvis was performed. The MRI protocol included T2-weighted high-resolution images in transversal and sagittal projections, diffusion-weighted images (b values 50 and 800), and T1-weighted images in transverse projection before and after the administration of a gadolinium-based contrast agent (4 ml of gadobutrol). Dynamic acquisition of T1-weighted sequences was also performed. Additionally, FDG uptake in the pelvis was obtained simultaneously within 15–20 min.

This was followed by a whole-body MRI examination using the Dixon volumetric interpolated breath-hold examination (VIBE) T1-weighted sequence method useful for calculating the attenuation correction of PET images. Four datasets of T1-weighted images were reconstructed for analysis. The acquisition time in each position was up to 4 min, and the PET acquisition was adjusted to match the MRI sequence’s total duration for that specific position.

### Image analysis

Using dedicated software (MM Oncology, SyngoVia; Siemens Healthineers), two radiologists experienced in hybrid imaging independently performed image analysis, and the measured values from both were averaged. MRI images were used to record three orthogonal distances of the cervical tumour (tumour size), followed by semiautomatic segmentation of the corresponding FDG lesion. Additional recorded parameters included SUVmax (maximum standard uptake value), SUVpeak (average SUV within a small, fixed-size region of interest [ROI] centred on a high-uptake region of the tumour), SUVmean (mean SUV value) and MTV (metabolic tumour volume). Total lesion glycolysis (TLG; TLG = MTV × SUVmean) was also calculated. A similar procedure was used to assess lymph nodes, whereby lymph nodes with SUVmax > 2.5 were considered metastases.

In addition, we also recorded the sum of the MTV of the primary tumour and pelvic and para-aortic lymph nodes (MTV-S) as well as the sum of TLG for the primary tumour and pelvic and para-aortic lymph nodes (TLG-S). Pretreatment parameters (pre-SUVmax, pre-SUVpeak, pre-SUVmean, pre-MTV, pre-MTV‑S, pre-TLG and pre-TLG-S) and treatment-specific parameters at week 5 (mid-SUVmax, mid-SUVmean, mid-SUVpeak, mid-MTV, mid-MTV‑S, mid-TLG and mid-TLG-S) were recorded. To measure the apparent diffusion coefficient (ADC), an ROI was manually placed within the region with the highest FDG uptake and maximal area, avoiding surrounding tissues.

Furthermore, we recorded absolute and relative (percentage) changes in all investigated parameters between pre- and mid-PET/MRI (∆ and ∆%), except for SUVmean and SUVpeak.

### Treatment response evaluation

**Fig. 1:**
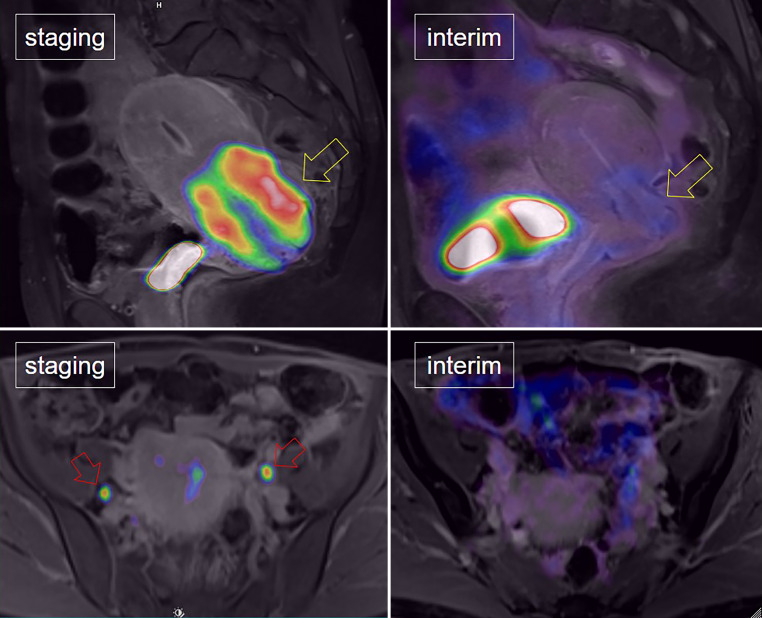
In a patient with cervical cancer, a complete metabolic response was observed. This was characterized by the complete disappearance of FDG uptake in both the primary cervical cancer lesion (yellow arrow) and the bilateral pelvic lymph node metastases (red arrows)

**Fig. 2:**
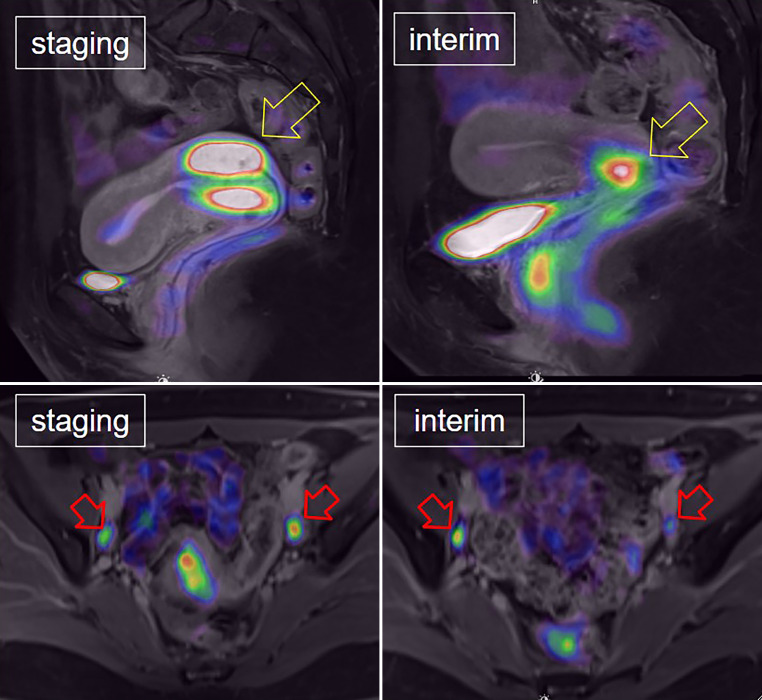
In a patient with cervical cancer, an incomplete metabolic response was observed. This was characterized by the persistence of FDG uptake in both the primary cervical cancer lesion (yellow arrow) and the bilateral pelvic lymph node metastases (red arrows)

The response to treatment evaluated by mid-PET/MRI (typically performed at week 5) was categorized into three types based on the RECIST 1.1 [16] and PERCIST [17] criteria:

1. *Interim complete metabolic response (ICMR)*: No morphological or metabolic evidence of cancer on mid-PET/MRI, regardless of initial staging (Fig. 1).
2. *Interim nodal response (INR)*: Absence of morphological and metabolic signs of nodal involvement and incomplete local remission, regardless of initial staging.
3. *Interim nodal persistence (INP)*: Persistent morphological or metabolic signs of nodal involvement after initial N1 or N2 findings and incomplete local remission (Fig. 2).

The combination of persistent nodal involvement and complete local remission at interim evaluation was not observed in the overall cohort.

Patients were then divided into two groups based on RECIST 1.1 and PERCIST criteria: those who achieved a complete metabolic response (CMR) and those who did not. CMR was defined as the absence of metabolically active lesions on post-PET/MRI performed 3 months after treatment, while any other finding was classified as non-CMR. These two groups were compared using metabolic, functional and morphological parameters obtained at pre-PET/MRI and mid-PET/MRI as well as using the absolute and relative changes in these parameters between the two examinations.

To evaluate correlations between the parameters of interest and survival outcomes, we used disease-free (DFS) and overall survival (OS). Local control (LC) was excluded due to only four cases of local recurrence in the entire cohort, thus rendering the results uninformative. DFS was defined as the period between treatment commencement and any relapse or death (both regarded as complete observations). Patients without relapse at the end of follow-up were censored. OS was defined as the duration from treatment initiation to death, with surviving patients censored at the last follow-up date.

In addition to the post-PET/MRI examination, our department conducts comprehensive gynaecological examinations, including pelvic ultrasound, at regular intervals of 3 to 6 months. Further investigations are only necessary if patients exhibit symptoms and there is substantial suspicion of disease recurrence.

### Statistical analyses

Standard frequency tables and descriptive statistics were used to characterize the patient sample. Because of significantly nonnormal distributions of most of the recorded variables, the differences between responders and nonresponders were tested for statistical significance using the Mann–Whitney U test.

The prediction potential of the recorded variables with respect to non-CMR was assessed using the receiver operating characteristic (ROC). The ROC curve, describing the observed sensitivity and specificity values across all possible threshold values within the range of a particular variable, was plotted for each variable. The area under the curve (AUC ROC) was then calculated to summarize the prediction factor in a single value, ranging from 1 (perfectly reliable prediction) to 0.5 (random guessing). Sensitivity and specificity were determined for specific threshold values. To assess the potential confounding of these predictors by TNM variables, the ROC analysis was repeated on a subsample uniform in terms of T and M (T2M0; 87 patients) in which the predictor values were also adjusted for the effect of N (i.e. divided by the mean value of the appropriate N category and then multiplied by the whole-sample mean).

Median follow-up time was determined using the inverse Kaplan–Meier method. The Kaplan–Meier survival curve estimation method along with the Gehan–Wilcoxon significance test was used to assess the associations of survival with categorical variables. The Cox proportional hazards model was used to explore the associations of continuous variables with survival. In order to visualise these associations with Kaplan–Meier plots, threshold values were determined for the continuous prognostic variables using an automated process. In this process, the Cox–Mantel *p*-values achieved after stratification of the sample into two groups by a particular continuous variable were calculated and plotted against all possible threshold values within the range of the variable, with a minimum group size of 10. Subsequently, the plots of *p*-value against threshold value were examined and a threshold value with good performance for both OS and DFS was determined for each continuous prognostic variable. In order to verify the independence of the prognostic variables, a multifactorial categorical Cox model was created for each of the major stratified predictors together with TNM factors and age.

All reported *p*-values are two tailed, and the level of statistical significance was set at α = 0.05. The STATISTICA data analysis software system (version 12; StatSoft, Inc. 2013. Tulsa, OK, US) was used; the ROC analysis, Cox proportional hazards model and threshold determination for survival predictors were implemented in MATLAB (released 2019a, The MathWorks, Inc., Natick, MA, USA).

## Results

### Treatment response

At mid-PET/MRI, 19 patients (17.6%) had an ICMR response and the remaining 89 patients (82.4%) had a partial response. According to stratification into different types of interim response, we observed ICMR, INR and INP responses in 19 (17.6%), 55 (50.9%) and 34 (31.5%) patients, respectively.

According to post-PET/MRI, the final response to treatment was classified as CMR (responders) in 82 patients (75.9%). In the remaining 26 patients (24.1%), the response was assessed as non-CMR (nonresponders). These nonresponders comprised 5 cases (4.6%) of significant partial remission with low residual metabolic activity, 7 cases (6.5%) of partial regression with persistent macroscopic residual tumour, 2 cases (1.9%) of local remission and partial remission in the area of lymphadenopathy, 11 cases (10.2%) of distant metastases and 1 case (0.9%) of no response at all (a persistent tumour).

A total of 35 patients (32.4%) were lost to follow-up. We know of 26 deaths (24.1%). Information for the remaining 9 patients (8.3%) is not available.

### Survival outcomes

Median follow-up was 49 months. The estimated 1‑, 2‑, 3‑, 4‑ and 5‑year DFS and OS rates for patients achieving CR (complete remission) and non-CR, for patients with different types of response, and for patients with < 5 or ≥ 5 cycles of chemotherapy used are summarised in Table 2.

**Table 2:** Survival outcomes in different groups of patients

Category	DFS					OS				
	1‑year	2‑year	3‑year	4‑year	5‑year	1‑year	2‑year	3‑year	4‑year	5‑year
*Response*										
CR	96.9% (93.1–100)	84.4% (76.4–92.5)	77.4% (67.5–87.2)	74.6% (64.1–85.2)	69.0% (56.6–81.4)	98.9% (96.7–100)	93.3% (87.7–99.0)	87.3% (79.4–95.3)	83.3% (73.9–92.7)	77.2% (65.2–89.2)
Non-CR	49.3% (29.9–68.6)	45.8% (26.1–65.6)	ND	ND	ND	90.9% (79.5–100)	69% (49.3–88.8)	ND	ND	ND
*Type of response*										
1	92.8% (81.6–100)	89.2% (75.6–100)	82.4% (64.4–100.4)	76.6% (53.3–99.8)	70.7% (42.3–99.1)	93.4% (82.6–100)	91.1% (78.6–100)	87.3% (71.2–100)	81.1% (58.6–100)	74.9% (46.0–100)
2	86.2% (77.1–95.4)	77.3% (66.0–88.6)	74.2% (62.2–86.3)	70.2% (57.2–83.2)	ND	98.2% (94.8–100)	91.5% (83.8–99.3)	85.6% (75.5–95.8)	79.6% (67.4–91.9)	70.6% (55.1–86.2)
3	70.7% (55.8–85.5)	58.1% (41.3–74.9)	ND	ND	ND	88.1% (77.5–98.7)	72% (56.3–87.7)	ND	ND	ND
*Cycles of ChT*										
< 5	78.9% (69.3–88.5)	68.4% (57.2–79.6)	58.3% (45.9–70.8)	55.0% (42.2–67.9)	49.4% (35.8–63.1)	93.1% (87.1–99.1)	81.3% (71.7–90.8)	70.6% (58.9–82.3)	65.6% (52.9–78.3)	59.3% (45.2–73.4)
≥ 5	89.4% (80.1–98.7)	82.4% (70.5–94.4)	79.5% (64.9–94.1)	76.5% (59.2–93.8)	ND	96.9% (91.7–100)	93.0% (83.6–100)	90.1% (77.0–100)	87.2% (70.4–100)	ND

*DFS* disease-free survival, *OS* overall survival, *CR* complete response, *ChT* chemotherapy, *ND* not defined

### Predictive potential of the assessed characteristics

**Fig. 3:**
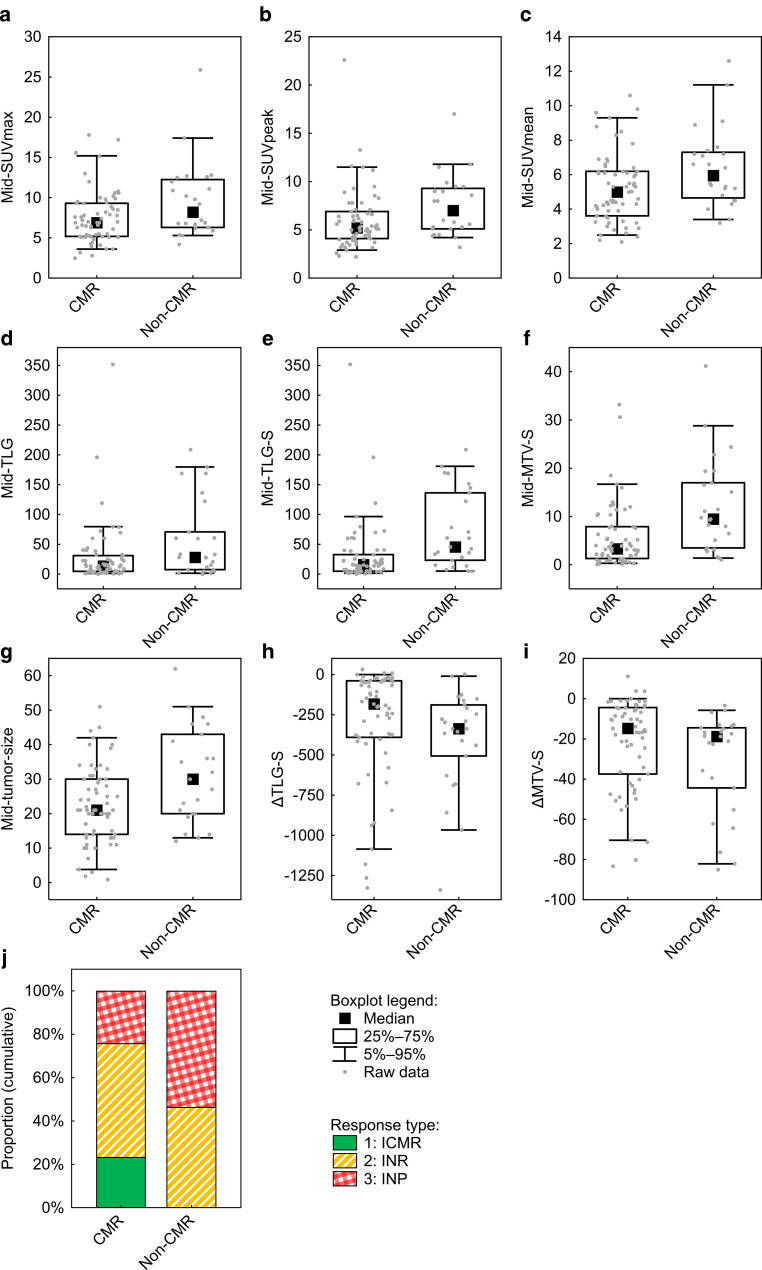
Comparison of the evaluated parameters between responders (complete metabolic response, *CMR*) and nonresponders (*non-CMR*), the differences in which reached statistical significance: **a** mid-SUVmax, **b** mid-SUVpeak, **c** mid-SUVmean, **d** mid-TLG, **e** mid-TLG‑S, **f** mid-MTV‑S, **g** mid-tumour size, **h** ∆TLG‑S, **i** ∆MTV‑S, **j** response type.* SUV* standardized uptake value, *TLG* total lesion glycolysis, *MTV* metabolic tumour volume, *S* sum, *∆* absolute change, *∆%* relative change

A statistically significant difference in the assessed parameters between responders and nonresponders was found for the following parameters: mid-SUVmax (*p* = 0.047), mid-SUVpeak (*p* = 0.016), mid-SUVmean (*p* = 0.020), mid-TLG (*p* = 0.033), mid-TLG‑S (*p* < 0.001), mid-MTV‑S (*p* = 0.001), mid-tumour size (*p* = 0.019), ∆TLG‑S (*p* = 0.033), ∆MTV‑S (*p* = 0.032) and response type (*p* = 0.002; Fig. 3).

**Fig. 4:**
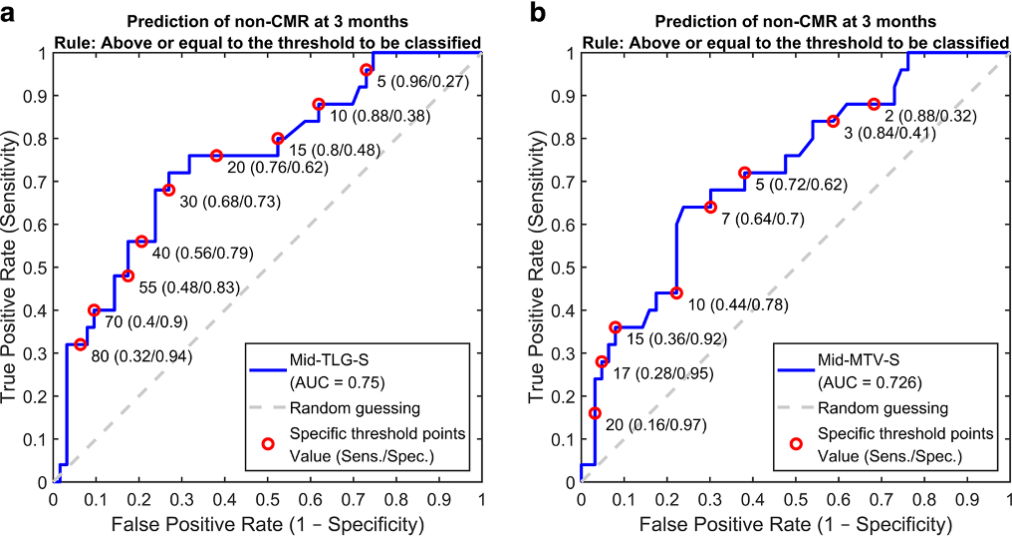
Area under the receiver operating characteristic curve (*AUC*) analysis of the evaluated parameters with good discriminatory ability for predicting failure to achieve a complete metabolic response (*non-CMR*). Illustrative threshold values are shown on the curves along with corresponding sensitivity and specificity values. The threshold values were set empirically as round values with reasonably uniform spacing along the curve. **a** mid-TLG‑S (sum of total lesion glycolysis for the primary tumour and pelvic and para-aortic lymph nodes), **b** mid-MTV‑S (sum of metabolic tumour volume of the primary tumour and pelvic and para-aortic lymph nodes)

**Fig. 5:**
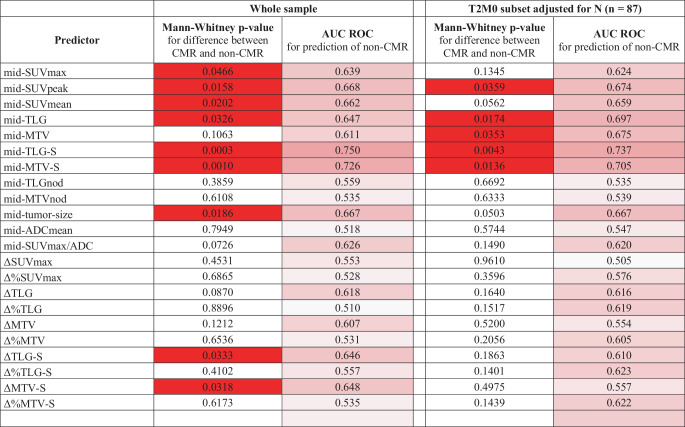
Area under the receiver operating characteristic curve (AUC ROC) values of parameters of interest for predicting failure to achieve a complete metabolic response (*non-CMR*). *P*-values significant at α = 0.05 are highlighted in solid red, ROC values are highlighted according to a scale from 0.5 (white) to 1 (red). *SUV* standardized uptake value, *TLG* total lesion glycolysis, *MTV* metabolic tumour volume, *ADC* apparent diffusion coefficient, *S* sum, *∆* absolute change

According to ROC analysis (Fig. 4), the mid-TLG‑S parameter showed the best discriminatory ability for predicting non-CMR (AUC ROC 0.750). The second factor with significant discriminatory ability was mid-MTV‑S (AUC ROC 0.726). These predictors remained statistically significantly different between responders and nonresponders and maintained their predictive ability even after repeating the analysis in a T2M0 subset of the sample and adjusting the predictor values for N (Fig. 5). The remaining parameters with whole-sample AUC ROC values lower than 0.69 did not appear to be good predictive factors.

The strongest factor, mid-TLG‑S, showed a sensitivity of 40% (95% confidence interval [CI] 21%–61%) and a specificity of 90% (CI 80%–96%) at a threshold value of 70.

Response type 2 (INR) or 3 (INP) predicted failure to achieve CMR with a sensitivity of 100% (95% CI 86.8%–100%) and a specificity of 23% (95% CI 14.6%–33.8%), while response type 3 (INP) achieved a sensitivity of 53.9% (95% CI 33.4%–73.4%) and a specificity of 75.6% (95% CI 64.9%–84.4%).

### Association of evaluated parameters with survival outcomes

**Fig. 6:**
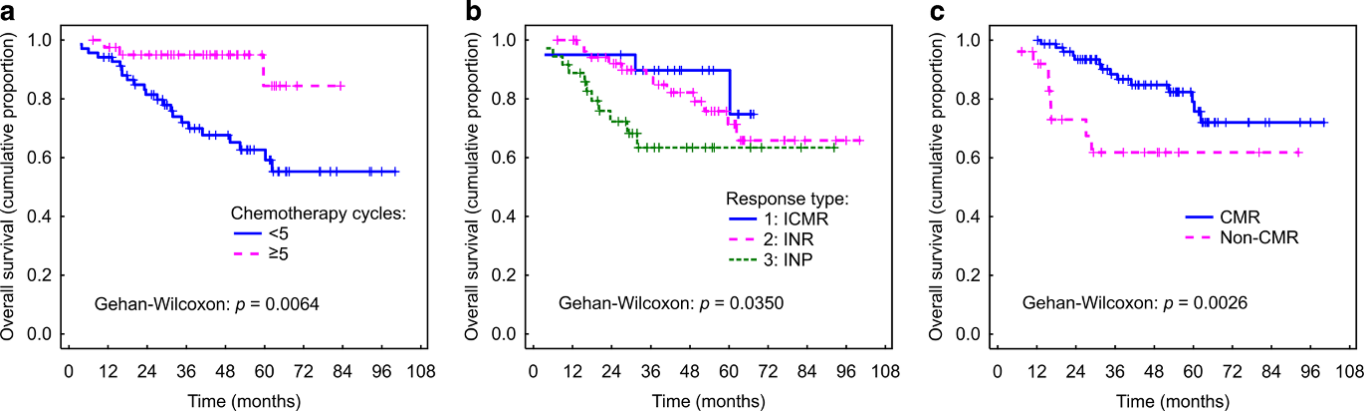
Statistically significant association of disease-free survival and overall survival with: **a** number of chemotherapy cycles, **b** response type, **c** complete metabolic response (*CMR*) vs. non-CMR on post-PET/MRI

We found a statistically significant association of DFS and OS with the following categorical and ordinal parameters: number of chemotherapy cycles < 5 cycles vs. ≥ 5 cycles (*p* = 0.020 and *p* = 0.006, respectively), response type 1 or 2 or 3 (*p* = 0.015 and *p* = 0.035, respectively), and CMR vs. non-CMR (*p* < 0.001 and *p* = 0.003, respectively; Fig. 6). Concerning continuous radiological variables, mid-MTV (*p* = 0.004 and *p* = 0.034, respectively), mid-TLG‑S (*p* = 0.001 and *p* = 0.018, respectively) and mid-MTV‑S (*p* = 0.001 and *p* = 0.009, respectively) were found to have a statistically significant prognostic value. For mid-MTV‑S, this prognostic ability was independent of TNM factors and age, as shown by the multifactorial categorical Cox model (Table 3).

**Table 3:** Multifactorial categorical Cox model for DFS and OS. Independent statistically significant prognostic factors are highlighted

		Model with mid-MTV				Model with mid-MTV‑S				Model with mid-TLG‑S			
		DFS		OS		DFS		OS		DFS		OS	
Factor/category		Relative risk (95% CI)	*p‑value*	Relative risk (95% CI)	*p‑value*	Relative risk (95% CI)	*p‑value*	Relative risk (95% CI)	*p‑value*	Relative risk (95% CI)	*p‑value*	Relative risk (95% CI)	*p‑value*
*Age*	< 60	1.000 (–)	–	1.000 (–)	–	1.000 (–)	–	1.000 (–)	–	1.000 (–)	–	1.000 (–)	–
	≥ 60	0.672 (0.316–1.429)	0.3016	1.214 (0.508–2.901)	0.6626	0.671 (0.314–1.432)	0.3023	1.175 (0.494–2.794)	0.7144	0.712 (0.324–1.563)	0.3965	1.276 (0.507–3.212)	0.6050
*T*	1	0.000 (–)^a^	0.9933	0.000 (–)^a^	0.9941	0.000 (–)^a^	0.9933	0.000 (–)^a^	0.9941	0.000 (–)^a^	0.9933	0.000 (–)^a^	0.9940
	2	1.000 (–)	–	1.000 (–)	–	1.000 (–)	–	1.000 (–)	–	1.000 (–)	–	1.000 (–)	–
	3	4.821 (1.959–11.862)	0.0006*	6.065 (2.111–17.428)	0.0008*	3.989 (1.591–10.005)	0.0032*	5.638 (1.976–16.087)	0.0012*	5.042 (2.080–12.223)	0.0003*	6.052 (2.094–17.489)	0.0009*
	4	0.000 (–)^a^	0.9961	0.000 (–)^a^	0.9969	0.000 (–)^a^	0.9961	0.000 (–)^a^	0.9970	0.000 (–)^a^	0.9961	0.000 (–)^a^	0.9969
*N*	0	1.000 (–)	–	1.000 (–)	–	1.000 (–)	–	1.000 (–)	–	1.000 (–)	–	1.000 (–)	–
	1	1.132 (0.449–2.855)	0.7921	1.197 (0.402–3.563)	0.7467	1.062 (0.423–2.665)	0.8987	1.034 (0.338–3.169)	0.9527	1.101 (0.435–2.787)	0.8393	1.206 (0.403–3.616)	0.7376
	2	1.482 (0.522–4.207)	0.4603	1.083 (0.285–4.111)	0.9069	1.784 (0.632–5.040)	0.2746	1.258 (0.353–4.484)	0.7239	1.396 (0.490–3.978)	0.5329	1.099 (0.291–4.156)	0.8890
*M*	0	1.000 (–)	–	1.000 (–)	–	1.000 (–)	–	1.000 (–)	–	1.000 (–)	–	1.000 (–)	–
	1	1.334 (0.307–5.795)	0.7007	0.590 (0.064–5.405)	0.6403	0.705 (0.135–3.677)	0.6788	0.310 (0.029–3.314)	0.3329	1.365 (0.311–5.988)	0.6801	0.618 (0.067–5.681)	0.6704
*Mid-MTV*	< 11.5	1.000 (–)	–	1.000 (–)	–	–	–	–	–	–	–	–	–
	≥ 11.5	1.970 (0.789–4.917)	0.1462	1.877 (0.658–5.351)	0.2390	–	–	–	–	–	–	–	–
*Mid-MTV‑S*	< 17	–	–	–	–	1.000 (–)	–	1.000 (–)	–	–	–	–	–
	≥ 17	–	–	–	–	3.803 (1.261–11.468)	0.0177*	3.694 (1.130–12.079)	0.0306*	–	–	–	–
*Mid-TLG‑S*	< 70	–	–	–	–	–	–	–	–	1.000 (–)	–	1.000 (–)	–
	≥ 70	–	–	–	–	–	–	–	–	1.736 (0.695–4.336)	0.2372	1.632 (0.515–5.165)	0.4050

*MTV* metabolic tumour volume, *TLG* total lesion glycolysis, *S* sum, *DFS* disease-free survival, *OS* overall survival, *T* tumour, *N* node, *M* metastasis, *CI* confidence interval

^a^Small risk estimates with extremely wide confidence intervals due to small number of patients in the group

**Fig. 7:**
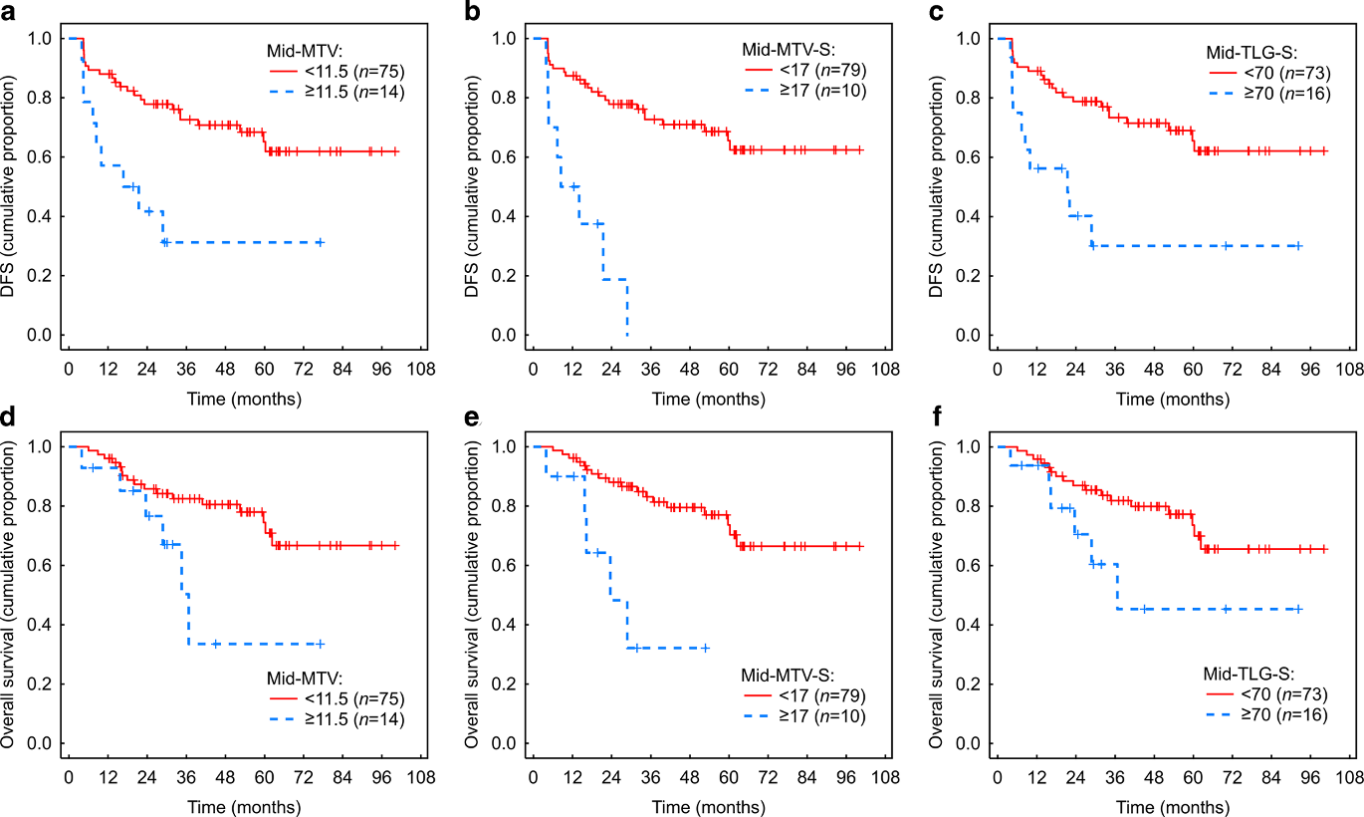
Treatment outcomes of patient groups separated by thresholds for significant parameters. Disease-free survival (*DFS*): **a** mid-MTV, **b** mid-MTV‑S, **c** mid-TLG‑S; overall survival (*OS*): **d** mid-MTV, **e** mid-MTV‑S, **f** mid-TLG‑S. *TLG* total lesion glycolysis, *MTV* metabolic tumour volume, *S* sum

For the continuous prognostic variables, threshold values providing a robust separation of patients with a high risk of short DFS and OS were determined using the semiautomatic process described in the methods, obtaining the following thresholds: mid-MTV: 11.5, mid-TLG-S: 70 and mid-MTV-S: 17 (Table 4 and Fig. 7).

**Table 4:** Thresholds separating the group of patients with a lower and higher probability of better DFS and OS

Category	DFS					OS				
	1‑year	2‑year	3‑year	4‑year	5‑year	1‑year	2‑year	3‑year	4‑year	5‑year
*Mid-MTV*										
< 11.5	87.1% (79.5–94.7)	77.7% (68.0–87.3)	72.0% (61.2–82.7)	69.2% (57.9–80.6)	63.3% (50.2–76.4)	95.3% (90.6–100)	85.4% (77.1–93.7)	81.6% (72.2–90.9)	79.0% (68.8–89.1)	72.4% (59.6–85.3)
≥ 11.5	54.9% (28.9–80.9)	38.0% (11.5–64.5)	ND	ND	ND	87.5% (70.2–100)	75.8% (52.2–99.5)	39.3% (4.0–74.5)	ND	ND
*Mid-TLG‑S*										
< 70	88.1% (80.7–95.5)	78.6% (69.0–88.2)	72.7% (61.9–83.5)	69.9% (58.4–81.3)	63.6% (50.1–77.0)	95.2% (90.3–100)	86.7% (78.7–94.7)	81.4% (71.9–90.9)	78.3% (67.8–88.8)	71.5% (58.3–84.7)
≥ 70	54.8% (30.3–79.3)	37.3% (11.7–62.9)	ND	ND	ND	88.8% (73.1–100)	69.7% (44.7–94.7)	46.7% (14.0–79.4)	ND	ND
*Mid-MTV‑S*										
< 17	86.5% (79.0–94.1)	77.7% (68.3–87.0)	72.1% (61.6–82.6)	69.4% (58.4–80.5)	63.7% (51.0–76.5)	95.6% (91.1–100)	87.7% (80.3–95.2)	82.0% (72.9–91.1)	78.0% (67.7–88.2)	71.7% (59.1–84.4)
≥ 17	42.0% (10.7–73.3)	12.2% (7.6–31.9)	ND	ND	ND	81.2% (56.0–100)	46.9% (10.2–83.6)	ND	ND	ND

*DFS* disease-free survival, *OS* overall survival, *MTV* metabolic tumour volume, *TLG* total lesion glycolysis, *S* sum, *ND* not defined

## Discussion

One current effort in the field of LACC is to identify patients who have a high likelihood of relapse after CCRT and who may benefit from treatment intensification, particularly in terms of adjuvant ChT. Recently, the long-awaited results of the OUTBACK trial, a randomised phase III trial investigating the effect of adjuvant administration of four cycles of paclitaxel/carboplatin in a 3-week regimen after standard CCRT with weekly cisplatin administration, were published [18]. Unfortunately, adjuvant ChT did not improve OS or PFS: OS at 5 years was similar in patients treated with adjuvant ChT compared to the control group without adjuvant ChT (72% vs. 71%; *p* = 0.91), and PFS at 5 years was also similar in patients treated with adjuvant ChT compared to the control group (63% vs. 61%, *p* = 0.61). Efforts to intensify treatment failed. However, the administration of adjuvant ChT was not individualised based on predicting the risk of disease persistence or early recurrence. This was therefore a nonstratified population.

The current work builds upon our previous research published in 2021 [6], which confirmed the assumption that when using PET/MRI scans during radical CCRT, there are metabolic morphological parameters that have discriminatory power for predicting non-CMR. In this previous study, these parameters were mid-MTV‑S (the best discriminator), mid-MTV, mid-tumour size, mid-TLG‑S, mid-TLG and ∆%SUVmax. Unfortunately, this study did not include a long-term comparison of treatment outcomes in relation to the very early treatment response (i.e. by mid-PET/MRI). It has already been shown in the literature that the early tumour response to treatment is an important predictor of disease recurrence, with significant prognostic significance for OS [19, 20]. Patients who achieve CMR after treatment have significantly better OS than those who do not [20, 21]. However, there is always a degree of subjectivity in the assessment of treatment response by any imaging modality, and it is therefore important to use highly sensitive and specific methods, especially when pathological verification of findings after CCRT is not possible. At our institution, long and systematic use of PET/MRI scans (mid-PET/MRI and post-PET/MRI) allows us to assess the response during and after treatment, mainly to maintain the ease of comparison between individual findings.

Although some parameters of posttreatment imaging have been shown to be effective predictors of OS, they do not allow for early and significant adjustment of treatment [22]. Information on the likelihood of disease persistence or early relapse should be obtained just before the decision on treatment intensification is to be made, i.e. optimally just before the end of treatment. Schernberg et al. [23] have convincingly shown that treatment response, as assessed by MRI scans performed during treatment, is strongly associated with OS. Patients in their cohort who achieved CR during treatment had fewer relapses, regardless of the initial disease stage. It has also been previously described that mid-treatment PET/CT can be used to assess the early response to treatment, allowing for a possible modification of treatment [24, 25].

Already in 2013, Oh et al. [26] evaluated the predictability of CMR achievement using PET/CT performed during the treatment (at week 4), but only on the basis of ∆%SUVmax compared to pretreatment PET/CT in 60 LACC patients treated with CCRT. They showed that ∆%SUVmax could predict CR on posttreatment PET/CT with a cut-off value of 59.7%. Xu et al. [27], who used a similar approach to ours, i.e. PET/MRI before, during (at the end of the third week) and after treatment in 41 LACC patients treated with CCRT, found that the combination of pre-MTV‑S, ∆%SUVmax and the percentage change in mean diffusion coefficient (∆%Dmean) during CCRT had the strongest predictive potential (AUC 0.912; *p* < 0.05). Gao et al. [28] evaluated the early response to treatment and also attempted to predict tumour recurrence using pre- and mid-PET/MRI (at week 4) in 51 patients with LACC. They evaluated pre- and mid-PET parameters (SUVmax, MTV, TLG) and MRI parameters (D = slow diffusion coefficient, F = perfusion-related diffusion fraction, D* = fast diffusion coefficient) and their percentage changes. Values of pre-SUVmax (*p* = 0.033), ∆%SUVmax (*p* = 0.032), mean TLG (*p* = 0.048), ∆%D (*p* = 0.009) and ∆%F (*p* = 0.008) were associated with the degree of early tumour shrinkage. Pre-SUVmax (*p* = 0.003), pre-TLG (*p* = 0.002), mid-TLG (*p* = 0.017), ∆%D (*p* = 0.011), nodal involvement (*p* = 0.001) and early tumour shrinkage rate (*p* = 0.035) were associated with recurrence-free survival.

Our current work has significantly expanded our previous dataset, both quantitatively and qualitatively, by comparing it with treatment outcomes at longer follow-up. The timing of mid-PET/MRI was still chosen to be at the end of week 5, as BT is initiated at week 6. Prior to BT application, it is advisable to obtain information on the current anatomical conditions in the pelvis as well as information on the early treatment response. In the current cohort of patients, non-CMR was significantly predicted by mid-MTV, mid-TLG, mid-TLG‑S, mid-MTV‑S, mid-tumour size and ∆%SUVmax, with only mid-MTV‑S and mid-TLG‑S having substantial discriminatory power for predicting non-CMR, which was also shown to be independent of TNM factors. Looking at the list of parameters with discriminatory power for predicting non-CMR across the available literature, these two parameters are the common denominator, with mid-TLG‑S currently the strongest parameter. Both parameters were also associated with treatment outcomes in terms of DFS and OS. For the mid-TLG‑S parameter, we were also able to identify a threshold that separates lower and higher probabilities of better DFS and OS, namely a value of 70, which achieves a specificity of 90% when used for the prediction of non-CMR. This aspect represents an important contribution to the discussion regarding the possible stratification of patients for subsequent adjuvant treatment.

The Swedish authors Strandberg et al. [29] attempted to find an association between selected parameters from pretreatment and very early after treatment commencement PET/MRI scans, i.e. after 1 week of treatment, and the prediction of OS. In a modest cohort of 11 patients, they reported only two relapses with a median follow-up of 24 months, and the baseline parameters functional tumour volume (FTV; analogous parameter in our study = pre-MTV), ∆FTV (analogous to our ∆MTV) and ∆ADC emerged as potentially predictive in their study.

Radiomic analysis methods also attempt to stratify the risk of LACC recurrence. Radiomics is an emerging field in which various features are extracted from quantitative medical images using different techniques. Radiomic features can quantify the intensity, shape and heterogeneity (texture) of a tumour and have been used for cancer detection, diagnosis, treatment response and prognosis. These radiomic features are automatically calculated from tumour ROIs [30]. Lucia et al. [31, 32] described a radiomic model based on PET/CT and MRI to predict recurrence in advanced cervical cancer. The predictive accuracy was 90% with radiomic modelling compared to 56–60% with standard clinical variables.

Recently, deep learning has demonstrated its superiority over conventional radiomic methods based on hand-crafted features [33]. Compared to conventional radiomic methods, deep learning simplifies the multistep procedure by automatically learning useful features from images and shows better predictive significance.

The randomised phase III GCIG INTERLACE trial [34], the results of which were presented at the European Society for Medical Oncology (ESMO) 2023 conference, also sought to intensify the treatment approach by adding induction ChT (IChT) and investigated induction with 6 weekly cycles of paclitaxel/carboplatin followed by CCRT with weekly cisplatin. The trial enrolled 500 patients, and median follow-up was 64 months. The 5‑year PFS rate was 73% with IChT and 64% with CCRT alone (*p* = 0.013). The corresponding 5‑year OS rates were 80% and 72% (*p* = 0.04). These are promising results but were again obtained in an unselected patient population.

Given the promising results of preclinical and clinical studies regarding the synergistic effect of RT and immunomodulatory drugs, a systematic evaluation of this combination in metastatic cervical cancer or LACC is warranted [35]. The ATEZOLACC trial is a multicentre randomised phase II trial prospectively testing the addition of the programmed death ligand 1 (PD-L1) inhibitor atezolizumab to enhance tumour immunogenicity in patients undergoing CCRT with cisplatin. Atezolizumab is administered concurrently with RT and then adjuvantly after RT in the study arm [36].

There are some limitations to the current study. The sample size was relatively small, and longer follow-up is needed to monitor the long-term survival outcomes. Despite the ability to find predictors with a high discriminatory ability for predicting non-CMR, we are still searching for an optimal threshold with high sensitivity and specificity.

The common feature of the treatment intensification studies described above is that they were applied to an unselected patient population. Our aim is to complement these approaches by finding an effective and reliable stratification tool to select patients for whom such intensification should be indicated. Our current study, despite the limitations mentioned above, suggests that the mid-TLG‑S and mid-MTV‑S indices (and especially their well-defined thresholds) may be good future candidates. However, validation in a larger sample and, above all, analysis of their transferability and the consistency of their success rates in different sites is a prerequisite.

Application of the semiautomatic segmentation tool to the PET-positive lesions could be challenging due to the close relations to surrounding organs with high FDG uptake (i.e. bladder or ureter), and precise confirmation by imaging specialists is necessary. However, we are convinced that the method of analysis is repeatable and transferable.

## Conclusion

In this study, we confirmed that the use of PET/MRI during CCRT can lead to identification of metabolic parameters with discriminatory ability for predicting non-CMR, namely mid-MTV‑S and mid-TLG‑S. We were also able to identify thresholds for these parameters, which can be used to identify patients who are more likely to have worse DFS and OS. The type of early response during CCRT is also significantly associated with DFS and OS. These aspects are an important contribution to the discussion regarding the possible stratification of patients for subsequent individualised adjuvant treatment.
